# Integrated clinical and prognostic analyses of mTOR/Hippo pathway core genes in hepatocellular carcinoma

**DOI:** 10.1007/s13105-024-01015-0

**Published:** 2024-03-12

**Authors:** Tianhang Feng, Ping Chen, Tao Wang, Chunyou Lai, Yutong Yao

**Affiliations:** 1 Department of Hepatobiliary and Pancreatic Surgery, Sichuan Academy of Medical Sciences, Sichuan Provincial People’s Hospital, School of Medicine, University of Electronic Science and Technology of China, Chengdu, China; 2 Department of Laboratory Medicine, West China Second University Hospital, Sichuan University, Chengdu, China

**Keywords:** Hepatocellular carcinoma, mTOR, Hippo, Prognosis, Gene signature

## Abstract

Hepatocellular carcinoma (HCC) is one of the most aggressive and dismal cancers globally. Emerging evidence has established that mTOR and Hippo pathways are oncogenic drivers of HCC. However, the prognostic value of these pathways in HCC remains unclear. In this study, we aimed to develop a gene signature utilizing the mTOR/Hippo genes for HCC prognostication. A multiple stage strategy was employed to screen, and a 12-gene signature based on mTOR/Hippo pathways was constructed to predict the prognosis of HCC patients. The risk scores calculated by the signature were inversely correlated with patient prognosis. Validation of the signature in independent cohort confirmed its predictive power. Further analysis revealed molecular differences between high and low-risk groups at genomic, transcriptomic, and protein-interactive levels. Moreover, immune infiltration analysis revealed an immunosuppressive state in the high-risk group. Finally, the gene signature could predict the sensitivity to current chemotherapeutic drugs. This study demonstrated that combinatorial mTOR/Hippo gene signature was a robust and independent prognostic tool for survival prediction of HCC. Our findings not only provide novel insights for the molecular understandings of mTOR/Hippo pathways in HCC, but also have important clinical implications for guiding therapeutic strategies.

**Supplementary Information:**

The online version contains supplementary material available at 10.1007/s13105-024-01015-0.

## Introduction

Hepatocellular carcinoma (HCC) is the most common type of liver cancer and accounts for 75–90% of all liver cancers, causing over 600,000 deaths annually [24, 28]. Despite significant advancements in diagnosis and treatment, the 5-year survival rate of HCC patients remains low, with only ~ 18% of patients surviving beyond 5 years after diagnosis [22]. Although drugs like sorafenib and regorafenib have shown some effectiveness against advanced HCC, their overall impact on survival is limited [13, 17]. Actually, one of the major challenges in treating HCC is the tumor heterogeneity and high frequency of recurrence, which makes it difficult to predict the outcome and design effective treatments [7, 9]. Therefore, there is an urgent need for better predictive tools to identify patients at high risk of recurrence and poor outcomes, in order to develop more targeted and effective therapies.

Conventional prognostic models for HCC rely on clinicopathological factors including tumor size, number of lesions, microvascular invasion, and cirrhosis, as well as the levels of certain biomarkers in serum [4, 14, 25]. However, these prognostic models have limited sensitivity and specificity and do not support the identification of meaningful patterns of prognosis, particularly given the substantial heterogeneity of HCC. To address this challenge, multigene classifiers have been proposed as a promising solution to predict the outcome and recurrence of HCC, but their clinical utility remains limited [15, 18]. Therefore, there is a pressing demand for the development of novel molecular tools that can facilitate the accurate prediction of HCC prognosis.

The mammalian target of rapamycin (mTOR) and Hippo signaling pathways are extensively identified as two important drivers of HCC. mTOR pathway is a highly conserved serine/threonine kinase signaling pathway that plays a crucial role in the regulation of cell growth [2], and hepatic metabolisms [12]. Dysregulation of mTOR pathway has been proved to contribute to the development and progression of the disease [27]. In HCC, activation of mTOR leads to increased cell proliferation and survival, angiogenesis, and decreased apoptosis, all of which contribute to tumor growth and metastasis [30]. On the other hand, Hippo is a conserved signaling that controls cell proliferation, survival, and differentiation in various tissues [21]. Hippo pathway has been shown to play an important role in HCC, controlling cell proliferation, survival, angiogenesis, and inflammation [29].

Interestingly, recent studies have uncovered a significant interplay between the mTOR and Hippo pathways in the progression of HCC [3]. Investigations revealed that these pathways interact to regulate key cellular processes. Specifically, LATS kinase in Hippo pathway attenuates mTORC1 activation by impairing the interaction of Raptor with Rheb [8]. Likewise, YAP can activate mTORC1 activity and thus regulates lipid metabolism [26]. Reciprocally, mTOR complexes (including mTORC1 and mTORC2) controls YAP activity and downstream cell proliferation [1]. As such, the integration of molecular signature analyses of mTOR-Hippo signaling may hold potential for the development of a prognostic prediction model for HCC, offering clinicians a powerful tool for assessing patient outcomes and guiding personalized therapeutic strategies.

In this study, we comprehensively investigated the expression patterns of Hippo-YAP signaling genes using transcriptomic data from public datasets. We constructed a prognostic model based on the expression of Hippo-YAP signaling genes for predicting the survival, immune infiltration, and chemotherapeutic sensitivity of patients with OSCC.

## Methods

The RNA expression data and clinical information of HCC used in this study were acquired from TCGA portal (https://portal.gdc.cancer.gov/) and ICGC database (http://dcc.icgc.org), respectively. The training set consisted of RNA-seq data from 374 HCC patients obtained from TCGA, while the testing sets comprised RNA-seq data from ICGC (*n* = 240) obtained from ICGC database. The data for this comprehensive set of genes from mTOR and Hippo pathways were sourced from PathCards database (https://pathcards.genecards.org/). A comprehensive description of methods can be found in Supplementary Materials.

## Results

### Functional enrichment analysis related to mTOR/Hippo pathway genes in HCC

**Fig. 1:**
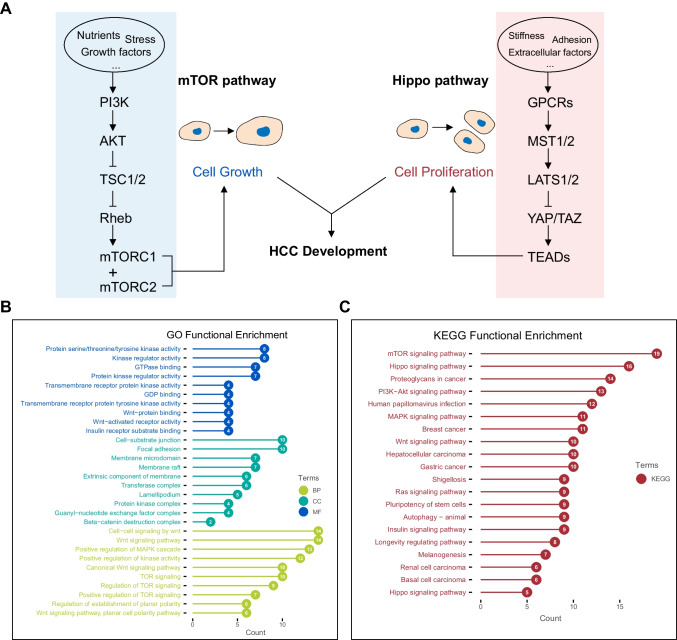
Functional enrichment analysis of the mTOR/Hippo genes. **A** A diagram showing the synergistical effect of mTOR and Hippo pathways in HCC. **B**–**C** GO (**B**) and KEGG (**C**) analysis showing the functional enrichment of enrolled genes in mTOR and Hippo pathways

mTOR pathway senses nutrients and controls cell proliferation, while Hippo pathway senses mechanical signals and regulates cell proliferation. Recent studies suggest that the two pathways exhibit crosstalk in HCC, and aberrations in these pathways contribute to HCC growth (Fig. 1A). To identify potential biological functions of mTOR/Hippo genes, we conducted functional enrichment analyses using Gene Ontology (GO) and Kyoto Encyclopedia of Genes and Genomes (KEGG) databases. GO analysis revealed significant enrichment of terms related to protein kinase activity, GTPase binding, and focal adhesions (Fig. 1B). KEGG analysis showed enriched pathways involved in mTOR and Hippo signalings, human cancers, and stem cell pluripotency (Fig. 1C).

### Establishment and validation of the mTOR/Hippo gene signature of HCC prognosis

**Fig. 2:**
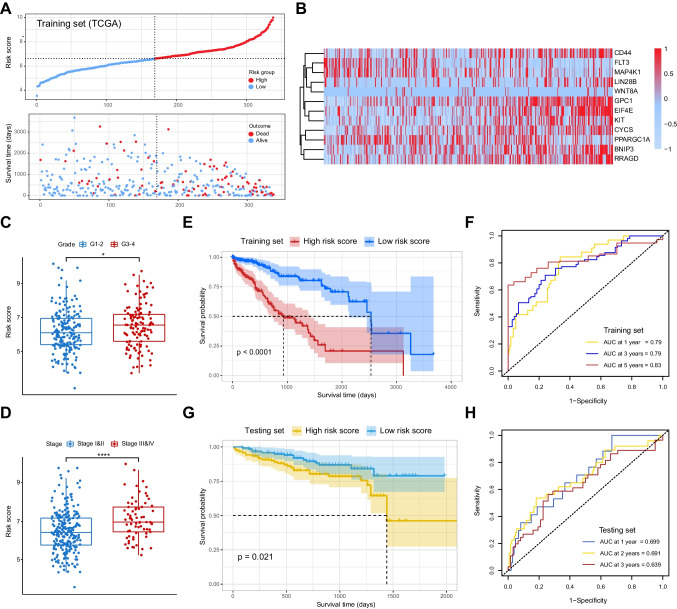
Construction and validation of the mTOR/Hippo gene signature. **A-B** Diagrams showing the 12 mTOR/Hippo risk score genes in HCC cohort from TCGA. **C**-**D** Diagrams showing the correlations between risk score and HCC grades (**C**) and stages (**D**). **E** Kaplan–Meier analysis showing the OS in different risk score groups in TCGA cohort. **F** The ROC curves showing the predictive efficiency of the model in TCGA cohort. **G** Kaplan–Meier analysis showing the OS in different risk score groups in ICGC cohort. **F** The ROC curves showing the predictive efficiency of the model in ICGC cohort

To construct a risk model based on mTOR/Hippo genes for predicting the prognosis of HCC patients, we identified a risk model consisting of 12 genes, including CD44, FLT3, MAP4K1, LIN28B, WNT8A, GPC1, EIF4E, KIT, CYCS, PPARGC1A, BNIP3 and RRAGD (Figs. 2A–B and S1A–B). The risk score was tightly associated with WHO grades and histological stages of HCC patients, but not related to patient genders and ages (Figs. 2C–D and S1E–F). To evaluate the prognostic significance of this model, we performed Kaplan–Meier survival analysis and found that patients in the high-risk score group had a significantly lower survival rate compared to those in the low-risk score group (Fig. 2E). Time-dependent receiver operating characteristic (ROC) curves were used to assess the predictive performance of the risk signature. The area under the curve (AUC) values at 1, 3 and 5 years were 0.79, 0.79 and 0.83, respectively, indicating the risk signature with robust prognostic accuracy (Fig. 2F). To further explore the utility of the risk model in different subgroups of patients with HCC, we stratified patients based on age (< = 65 vs. > 65 years), gender (male vs. female), grade (G1-2 vs. G3-4), and stage (stage I-II vs. stage III-IV). Results showed that the high-risk score group consistently exhibited a higher proportion of patients with poor outcomes than the low-risk score group across all subgroups (Figure S2).

To validate the robustness of the established gene signature, we conducted further analysis using independent ICGC dataset. Similarly, patients from the validation cohort were classified into high- and low-risk groups based on their median risk score (Figure S1C–D). The Kaplan–Meier survival analysis and ROC curves demonstrated that patients in the low-risk group had better survival rates compared to those in the high-risk group (Fig. 2G–H). These findings further confirm the reliability and accuracy of the prognostic signature comprising 12 mTOR/Hippo genes, which may serve as a valuable tool for predicting outcomes in patients with HCC.

### mTOR/Hippo gene signature as an independent prognostic factor in HCC

**Fig. 3:**
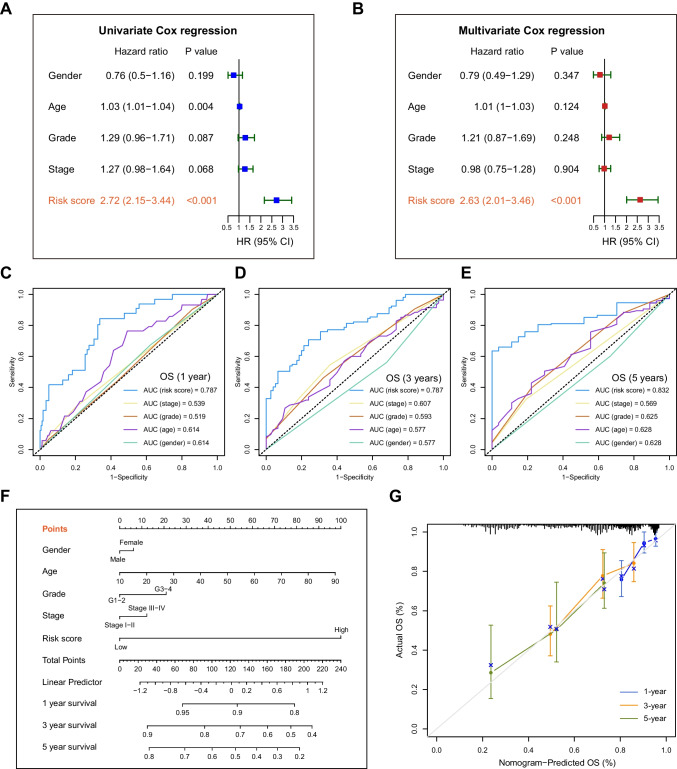
Independent prognostic significance of the mTOR/Hippo gene signature. **A**-**B** Univariate (**A**) and multivariate (**B**) Cox regression analysis showing that the gene signature was risk score was an independent predictor of OS in HCC patients in TCGA cohort. **C-E** The ROC curves of all the parameters in prediction OS of HCC patients in 1-(**C**), 3- (**D**) and 5-year (**E**). **F** A nomogram showing the predicting probabilities of the OS in HCC patients. **G** The calibration plots showing the accuracy of survival prediction. X-axis: predictive survival; Y-axis: actual survival

Next, we investigated whether the mTOR/Hippo gene signature was an independent prognostic factor in HCC, and performed univariate and multivariate Cox regression analyses, with covariates including patient gender, age, grade, stage, and risk scores. Results of the univariate analysis showed a statistically correlation of patient age and the risk score with the outcome (*p* < 0.05) (Fig. 3A). And the multivariate analysis showed a significant association only in the risk score with patient outcomes (*p* < 0.001) (Fig. 3B).

Moreover, it was observed that the risk score had the highest predictive power among all the clinical parameters across 1-, 3- and 5-year survival predictions (Fig. 3C–E). Then, we constructed a nomogram by combining independent prognostic factors (Fig. 3F). The calibration chart exhibited excellent consistency between the actual observation and predictive status for 1-, 3-, and 5-year OS (Fig. 3G), suggesting that the nomogram may serve as a clinically quantitative tool to predict the prognosis of HCC patients.

### Molecular characteristics of the mTOR/Hippo gene signature in HCC

**Fig. 4:**
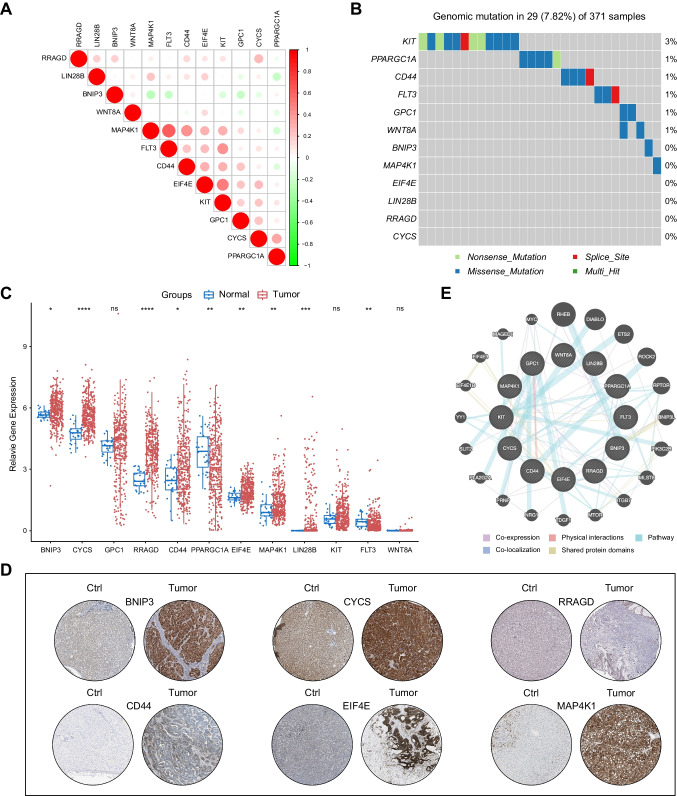
Molecular characteristics of the mTOR/Hippo gene signature. **A** The corrplot showing the correlations of the signature genes. **B** A waterfall plot showing the genomic alterations of the signature genes in TCGA cohort. **C** A chart showing the differentially expressions of the signature genes in HCC tissues and normal tissues. **D** IHC images showing the expressions of the signature genes in HCC samples and controls from HPA. **E** A network diagram showing the protein–protein interactions of the signature genes from GeneMANIA

To reveal the molecular characteristics of the mTOR/Hippo gene signature, we first examined the correlations of the signature genes. Results showed that several genes were highly correlated with others, including MAP4K1 with FLT3, MAP4K1 with CD44, FLT3 with KIT, EIF4E with KIT, and CYCS with PPARGC1A (Fig. 4A), suggesting that these genes may synergistically regulate HCC development. Next, we investigated the genomic alterations of signature genes. We found that the mutation rate of the signature genes was extremely low in HCC samples (< 3%), indicating that these genes trended to be genomically stable (Fig. 4B). Thus, we asked whether the expression levels of these genes were statistically different in HCC samples and normal controls. Results showed that the mRNA levels of BNIP3, CYCS, RRAGD, CD44, EIF4E, MAP4K1, and LIN28B were higher in tumor samples, whereas the mRNA levels of PPARGC1A and FLT3 were higher in normal samples (Fig. 4C). Therefore, we conclude that the distinct expressions of signature genes may not attribute to the genomic mutations, but be due to transcriptional regulations.

Then, we confirmed the protein levels of differentially expressed signature genes in HCC samples. It’s found that IHC signals of BNIP3, CYCS, RRAGD, CD44, EIF4E, and MAP4K1 were consistently high in tumor cells of HCC, compared with normal tissues (Fig. 4D), implying that these genes were indeed ectopically expressed in HCC. Finally, we assessed protein interactions of the signature genes using the PPI (protein–protein interaction) analysis. As displayed in the network architecture, the 12 mTOR/Hippo signature genes were surrounded by 20 hub genes. The large size of the circles in hub genes, including RHEB, DIABLO, ETS2, ROCK2, RPTOR, BNIP3L, PIK3C2B and MLS8 indicated high correlations of interactions (e.g., co-expression and physical interaction) (Fig. 4E). Altogether, these results suggest that the transcriptional regulation and protein interactive network, but not genomic alteration, are potential mechanisms by which the signature genes contribute to HCC development and prognosis determination.

### Immune landscape of the mTOR/Hippo gene signature in HCC

**Fig. 5:**
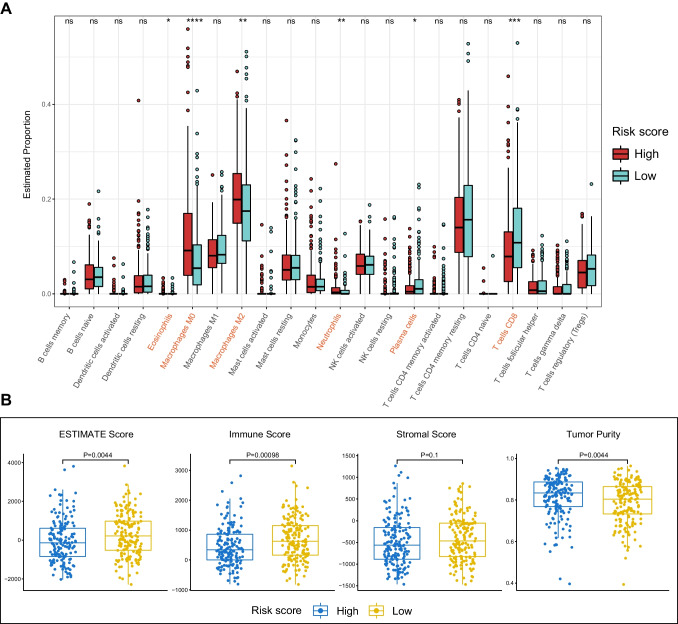
The immune landscape of the mTOR/Hippo gene signature. **A** Box plots showing the variation of immune cell populations across distinct risk score groups of HCC. **B** Diagrams showing the comparative analysis of ESTIMATE score, immune score, stromal score, and tumor purity among different risk score groups of HCC

Immune cell infiltration has been identified as a crucial factor in the prognosis of HCC. Thus, we evaluated whether there were distinct immune infiltration patterns among different risk score groups. The CIBERSORT algorithm was employed to estimate the proportion of 22 immune cell types in HCC samples (Fig. S3A). Results showed that the high-risk score group had higher ratio of several immune cells, including M0 and M2 macrophages and neutrophils, and lower ratio of plasma cells and CD8 T cells, indicating a more inactivated and immunosuppressive tumor microenvironment in the high-risk score group (Fig. 5A).

Furthermore, we employed the ESTIMATE algorithm to calculate the immune score, stromal score, tumor purity, and ESTIMATE score in different risk score subgroups. Results showed that the low-risk score group had higher ESTIMATE and immune scores, and lower tumor purity compared to the high-risk score group (Figs. 5B and S3B). This suggests that the poor prognosis of the high-risk score group may be partly attributed to the higher tumor purity. Overall, these results provide insights into the immune landscape of different risk score groups in HCC.

### Assessment of chemotherapeutic efficacy using the mTOR/Hippo gene signature

**Fig. 6:**
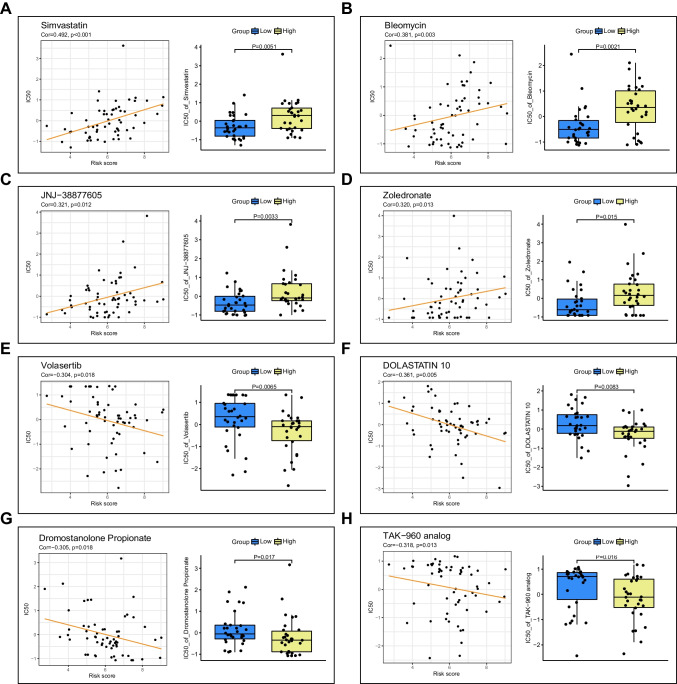
Prediction the drug sensitivity by the mTOR/Hippo gene signatures. **A**-**H** The scatter and box plots showing correlations between the gene signature and IC50 of multiple chemotherapeutic drugs

In order to provide clinical guidance of chemotherapeutic strategies in different risk score patients with HCC, we investigated the potential of the mTOR/Hippo gene signature to predict cell response to chemotherapy. Thereby, we assessed the sensitivity of different risk score groups to FDA-approved or clinical trial anti-cancer drugs using the CellMiner database, and analyzed the correlation between the risk score and the IC50 of each drug. We identified the top 8 drugs that exhibited distinct sensitivities between the low- and high-risk score groups. Notably, the IC50 of Simvastatin, Bleomycin, JNJ-38877605, and Zoledronate were observed to increase as the risk score increased, indicating that these drugs may be more effective in the low-risk score group** (**Fig. 6A–D). Conversely, the IC50 of Volasertib, Dolastatin 10, Dromostanolone Propionate, and TAK-960 analog were observed to decrease as the risk score increased, indicating that these drugs may be more suitable for the high-risk score group (Fig. 6E–H). These observations provide potential guidance for selecting chemotherapeutic strategies for HCC patients.

## Discussion

HCC is a widespread cancer type that exhibits uncontrollable aggressiveness and unfavorable prognosis [19]. Therefore, there is an urgent need for accurate and reliable prognostic prediction in HCC due to its high prevalence, aggressive nature, and poor prognosis. The malignancy of HCC primarily depends on signaling pathways, including mTOR and Hippo to drive the tumorigenic events [10]. In this study, we developed a robust model using 12 mTOR/Hippo gene to predict the prognosis of HCC patients. Our findings suggest that this model has potential to facilitate prognostic monitoring of HCC patients.

So far, numerous studies have elucidated the relationship between the mTOR/Hippo pathways and HCC, offering potential avenues for predicting prognosis using the mTOR/Hippo related genes. Compared to single-gene indicators, a set of genes can produce a more precise and reliable signature for prognostic prediction. To this end, we obtained gene expression profiles from the TCGA database and developed a robust mTOR/Hippo gene risk score model, which was effectively validated by independent cohort. Our 12-gene risk score model demonstrated an improved ability to predict patient prognosis compared to current clinical indicators. Our findings suggest that current prognostic factors, including gender, age, disease grade, histological stages, and TNM stages, are not reliable for outcome prediction (Fig. 3). Notably, our risk score model showed superior predictive ability for both the overall HCC cohort and subgroups. It’s noted that some pilot studies have identified prognostic models in HCC, including those utilizing either mTOR or Hippo genes [20], however, our work provides a more comprehensive and accurate model for prognostic estimation by combining these two pathways, deepening our understanding of gene set-based predictions.

Among all the 12 signature genes in the risk score model, we found that most of the genes were statistically different between tumor tissues and normal controls, and in particular, two genes (PPARGC1A and FLT3) were decreased in the tumor tissue (Fig. 4C). This notion was supported by the fact that these two genes were enriched in the low-risk score groups (Fig. 2A–B). PPARGC1A encodes a transcriptional coactivator well-known as PGC1-α, participating in mTOR-controlled mitochondrial metabolisms. Previous studies have demonstrated the lower PGC-1α expression in HCC compared to normal liver tissues, and knockdown of PGC-1α led to a cancerous phenotype with immature and de-differentiated morphology in HCC cells [16]. Here, we found that high PGC1-α expression was enriched in patients with low-risk scores, supporting the notion that PGC-1α may serve as a tumor-suppressor in HCC. Moreover, FLT3, encoding a class III receptor tyrosine kinase, exhibited reduced expression in a significant proportion of HCC patients, which was consistent with our findings that FLT3 was decreased in HCC tumor tissues [23]. On the contrary, other signature genes (e.g., EIF4E, MAP4K1) were shown to be highly expressed in tumor samples, supported by their functions in either mTOR or Hippo regulations. Additionally, RRAGD encodes a pivotal member of the Rag guanosine triphosphatases (GTPases) family, RagD, that assumes a critical role in the activation of mTORC1. RagD differentially defines substrate specificity downstream of mTORC1, promoting phosphorylation of lysosomal substrates TFEB/TFE3 [5]. This highlights RagD's significance in orchestrating mTORC1 regulation and underscores the functional diversity among Rag paralogues in mTORC1 activation [11]. Our results showed that RRAGD expression was distinct between tumor and control tissues, and elevated in the high-risk score groups, which indicates and contributes to the poor prognosis of HCC patients. Nevertheless, our study provides a comprehensive molecular landscape of HCC development by integrating mTOR and Hippo signaling pathways, and offers a reasonable explanation for the poor prognosis observed in the high-risk score group.

In clinical practice, the complex molecular network in HCC poses a challenge to the effectiveness of drug therapeutics and selections. Interestingly, our risk score model not only offers a tool for prognosis prediction but also enables the prediction of sensitivities to clinical chemotherapy drugs. For example, simvastatin, a cholesterol-lowering statin that prevents the synthesis of cholesterol, has been proved to inhibit HCC cell proliferation and increases the cell sensitivity to sorafenib [6]. However, it remains unclear which subgroup of HCC patients would derive the greatest benefit from simvastatin therapy. Here, we demonstrate that HCC patients with low-risk scores may benefit more from simvastatin treatment, as evidenced by the positive correlation between risk scores and the IC50 of simvastatin. Hence, our risk score model could be utilized to guide the development of chemotherapeutic regimens in clinical practice.

While our study primarily relies on bioinformatic analyses, it is essential to acknowledge the inherent limitation of our study. The findings presented in this study offer a potential scenario rather than definitive experimental evidence. The complexity of biological systems and the diverse regulatory mechanisms involved necessitate further experimental validations. Therefore, our study serves as a valuable foundation, providing hypothesis and insights that should be rigorously tested in future experimental settings to ensure robustness and clinical relevance.

## Conclusions

In conclusion, our study presents a novel mTOR/Hippo gene model for HCC that acts as a prognostic tool with significant implications for the immune infiltrate features and chemotherapy sensitivities. Our findings shed light on the prognosis of mTOR-Hippo signalings in HCC, offering molecular guidance for the improvement of HCC prognosis.

### Supplementary Information

Below is the link to the electronic supplementary material.

Supplementary file1 (DOCX 2935 KB)

## Data Availability

The gene expression datasets utilized and/or analyzed during the present study are publicly accessible on open repositories, specifically TCGA and ICGC. The analytic assays in this study are available from the corresponding author upon reasonable request.

## Abbreviations

HCC: Hepatocellular carcinoma
mTOR: Mammalian target of rapamycin
TCGA: The Cancer Genome Atlas
ICGC: International Cancer Genome Consortium
GO: Gene Ontology
KEGG: Kyoto Encyclopedia of Genes and Genomes
ROC: Receiver operating characteristic
AUC: Area under the ROC curve
PPI: Protein-protein interaction
