# Supplementary material for: Integrated clinical and prognostic analyses of mTOR/Hippo pathway core genes in hepatocellular carcinoma
Source: J Physiol Biochem. 2024 Mar 12;80(2):439–49. doi: 10.1007/s13105-024-01015-0 (PMC11074052; doi:10.1007/s13105-024-01015-0)
Supplement: Supplementary file 1 — Supplementary file1 (DOCX 2935 KB) [file 13105_2024_1015_MOESM1_ESM.docx]

**Supplementary Information of**

**Integrated Clinical and Prognostic Analyses of mTOR/Hippo Pathway Core Genes in Hepatocellular Carcinoma**

**Authors:** Tianhang Feng^1,#^, Ping Chen^2,#^, Tao Wang^1^, Chunyou Lai^1,*^, Yutong Yao^1,*^

**Affiliations:**

^1^ Department of Hepatobiliary and Pancreatic Surgery, Sichuan Academy of Medical Sciences, Sichuan Provincial People's Hospital, School of Medicine, University of Electronic Science and Technology of China, Chengdu, China.

^2^ Department of Laboratory Medicine, West China Second University Hospital, Sichuan University, Chengdu, China.

^#^These authors contributed equally to this work.

***Correspondence:** Chunyou Lai (laichunyou2010@163.com) and Yutong Yao (yaoyutong_meduestc@163.com)

**This file includes:**

Materials and Methods

Supplemental Figures 1-3

**Methods**

**Functional enrichment analysis**

To investigate biological functions and associated pathways of the mTOR/Hippo pathway genes identified in this study, Gene Ontology (GO) and Kyoto Encyclopedia of Genes and Genomes (KEGG) pathway enrichment analyses were performed using the "clusterprofiler" package of R software. These analyses were conducted to identify biological processes, molecular functions, cellular components, and pathways in which the genes were enriched. Specifically, the enriched GO terms and KEGG pathways were determined based on the false discovery rate (FDR) < 0.05.

**Development and validation** **of the prognostic gene signature**

To construct the gene signature of HCC, mRNA data from The Cancer Genome Atlas (TCGA) were set as the training cohort. First, univariate Cox regression analysis was performed with "survival" and "survminer" R packages. Next, LASSO-COX analysis using the "glmnet" R package was applied to remove genes that over-fitted with the model. Then, step-wise multivariate Cox regression analysis using "survival" and "survminer" R packages was conducted to determine the relationship between expression levels of genes and the OS of patients. A prognostic risk score formula was developed to calculate the probability of inferior survival for each sample. The relative regression coefficients (β) were used to multiply the optimal prognostic mRNA expression levels of specific genes.

Next, using the median risk score as a cut-off point, the HCC cohort was divided into high- and low-risk subgroups. Kaplan-Meier survival curves and time-dependent receiver operating characteristic (ROC) curves (analyzed using the “timeROC” R package) were generated to validate the prognostic significance of the risk score signature in predicting the OS. Finally, to evaluate the independent association between risk scores, clinical factors, and prognosis of HCC patients, univariate and multivariate Cox analyses were conducted, with P < 0.05 considered statistically significant. In order to evaluate the potential clinical relevance of the developed gene signature, a comprehensive nomogram was constructed by integrating the gene signature with clinicopathological factors, utilizing the "rms" package in R software. Calibration plots and ROC curves were generated to assess the accuracy and predictive ability of the nomogram. Finally, stratified analysis was performed to determine whether the gene signature could function as an independent prognostic factor. To verify the robustness of the prognostic signature, the same analyses were repeated in additional testing cohorts obtained from the ICGC (International Cancer Genome Consortium).

**Genomic and protein level analysis** **of the signature genes**

To investigate the mutational status of the signature genes, somatic mutation data of HCC was obtained from TCGA and visualized using the “maftools” R package. The mutation landscape was depicted using waterfall plots. At the protein level, the expression levels of signature genes that were found to be associated with prognosis in HCC were validated using the Human Protein Atlas (HPA) (https://www.proteinatlas.org/). The GeneMANIA website (http://genemania.org) was utilized to predict functionally related genes of the hub genes and construct a protein-protein interaction (PPI) network.

**Immune cell infiltration and tumor microenvironment analysis**

The infiltration levels of 22 immune cell types in HCC were estimated using RNA-seq data and the "CIBERSORT" R package, which uses gene expression profiles provided on the CIBERSORT platform. The p value for each sample deconvolution was determined globally by CIBERSORT, and only samples with p < 0.05 were selected for further analysis. To predict the tumor microenvironment (TME), immune scores, stromal scores, tumor purity, and estimated scores were calculated using the "ESTIMATE" R package.

**Correlation analysis of the risk score and sensitivity of chemotherapeutic drugs**

To explore associations of the gene signature with chemotherapeutic drugs, a correlation analysis was conducted to provide clinical guidance for risk score-based chemotherapy. The transcriptome of 60 cancer cell lines (NCI-60) and IC50 of over 20,000 compounds were obtained from CellMiner (https://discover.nci.nih.gov/cellminer/home.do), where all potential drugs were FDA-approved or under clinical trials. Pearson correlation analysis was employed to evaluate the correlation between the risk score and drug sensitivity, with statistically significant correlations determined at |cor|>0.3 and p<0.01. The scatter plot was generated using the “ggplot2” R package to display the significant correlations.

**Statistical analysis**

All statistical analyses and visualizations were carried out using the R software version 4.0.3 (https://cran.r-project.org/). A p-value of less than 0.05 was considered statistically significant.

**Supplementary Figures**


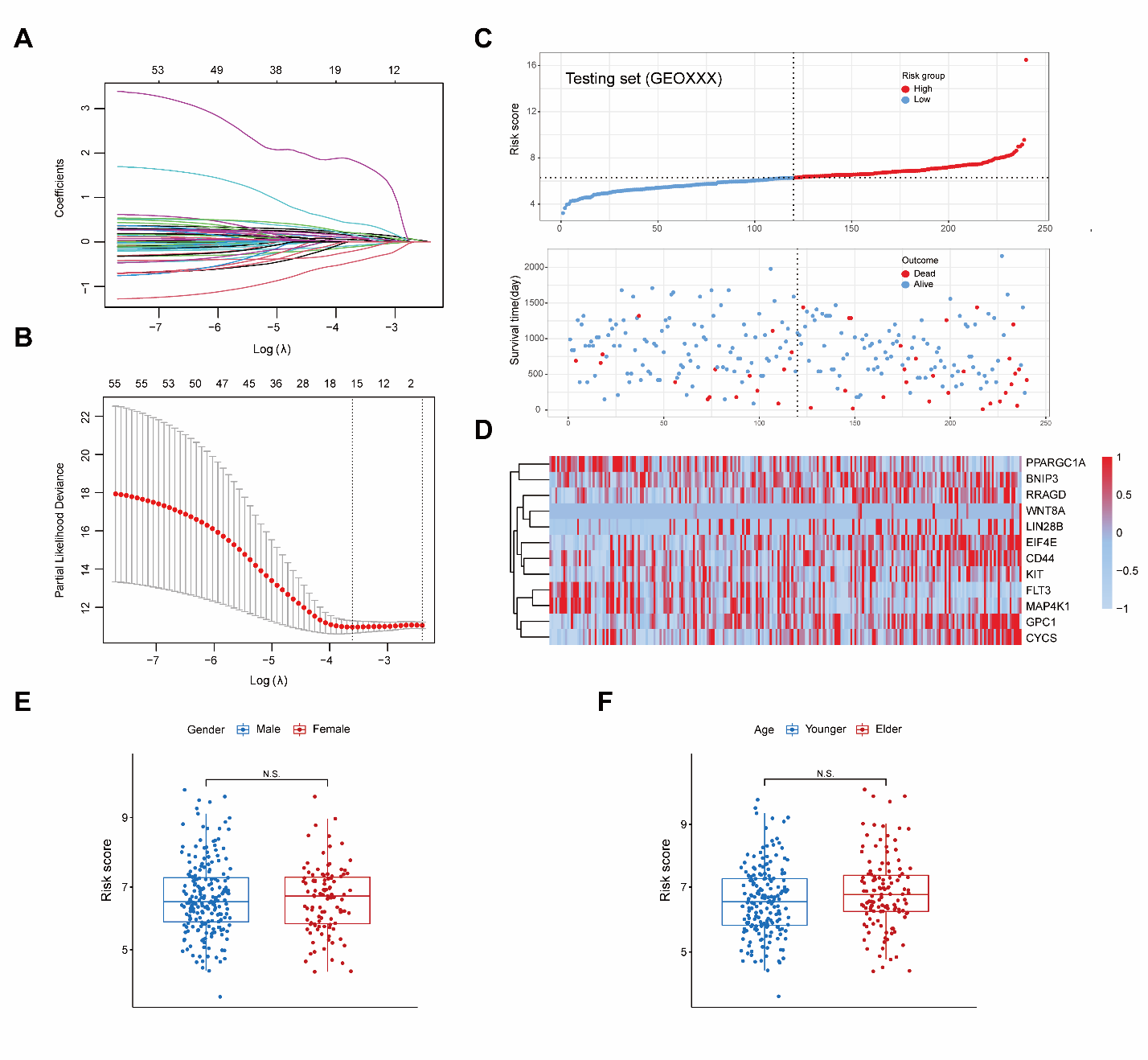
**Figure S1. Construction and validation of the mTOR/Hippo gene signature; related to Figure 2.**

**(A-B)** The procedure of the construction of gene signature prediction model using LASSO Cox regression analysis.

**(C-D)** Diagrams showing the 12 mTOR/Hippo risk score genes in HCC cohort from ICGC.

**(E-F)** Diagrams showing no significant correlations between risk score and patient genders (E) and ages (F) in HCC patients.


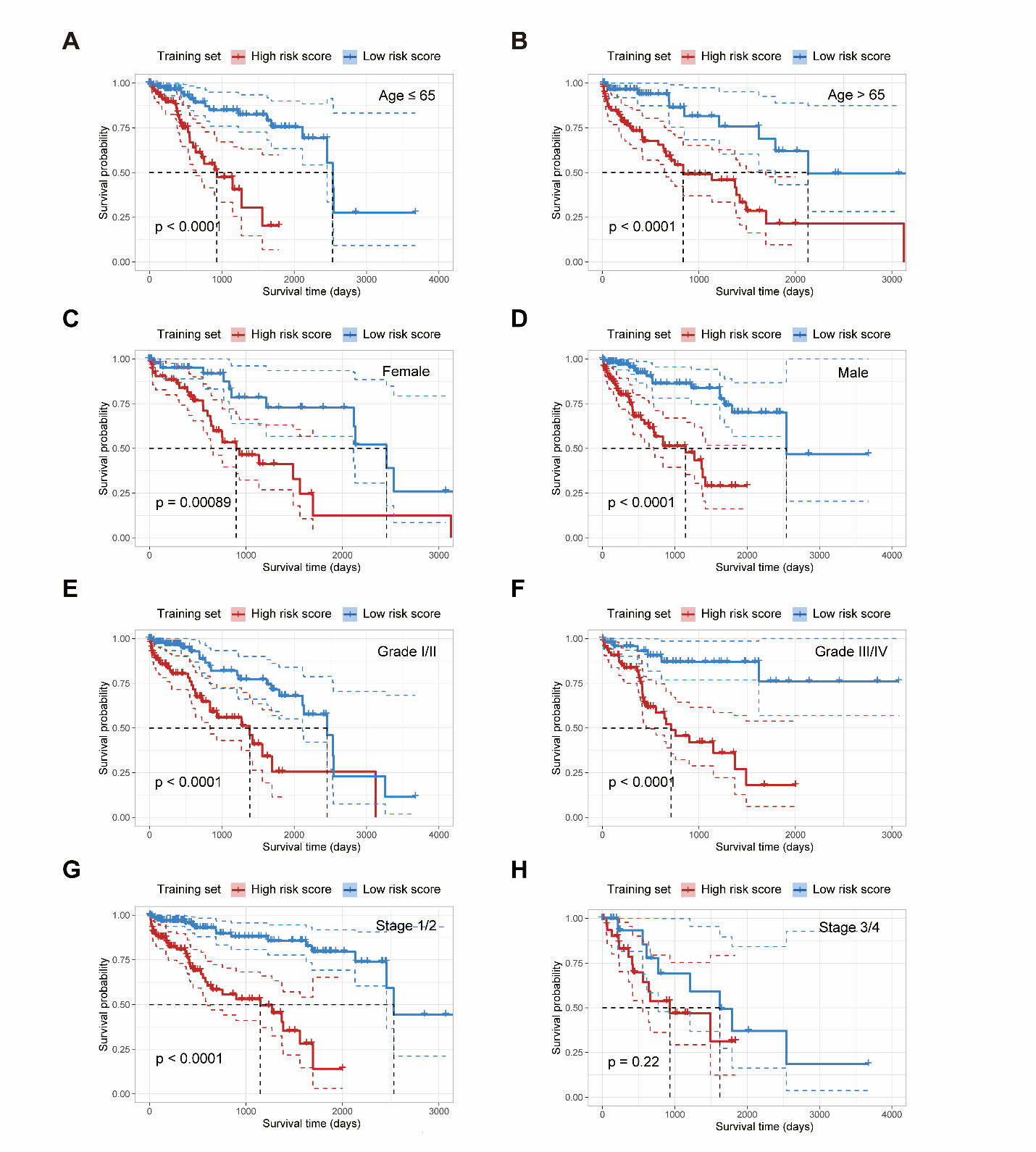
**Figure S2. Analysis of the mTOR/Hippo gene signature for survival evaluation in HCC subgroups; related to Figure 2.**

**(A-H)** Survival analysis showing that, across a range of sub-groups, including young (age≤65, A), old (age>65, B), female (C), male (D), WHO grade 1/2 (E), grade 3/4 (F), histological stage 1/2 (G), and except stage 3/4 (H), the high-risk score group was associated with inferior OS.


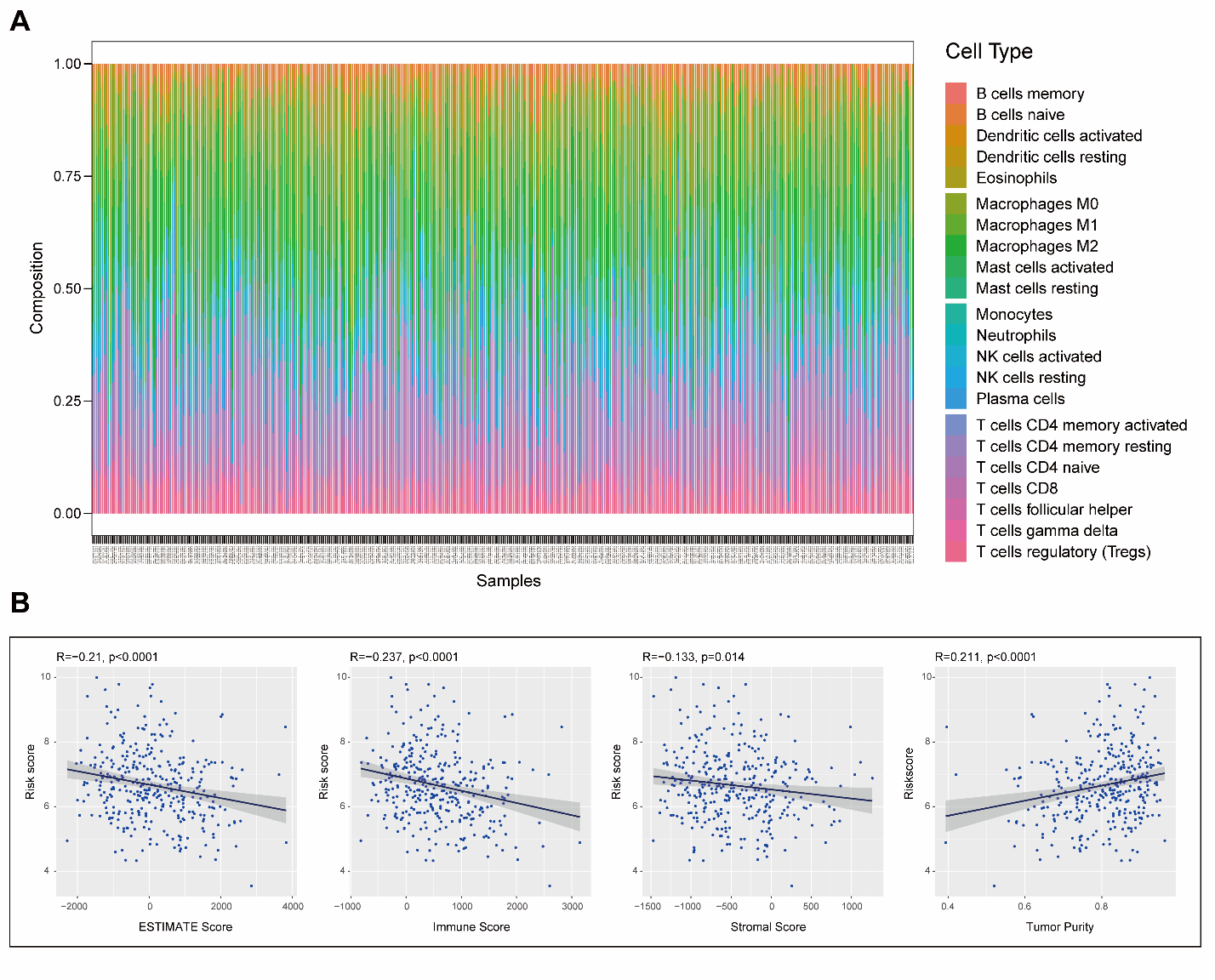
**Figure S3. Immune landscape of the mTOR/Hippo gene signature; related to Figure 5.**

**(A)** A diagram showing the differences in infiltrating immune cell compositions in HCC samples (TCGA). The bars of varying lengths in each sample indicate the proportions of distinct immune cells.

**(B)** Correlations of the risk score with ESTIMATE score, immune score, stromal score, and tumor purity in HCC samples (TCGA).
